# Aflatoxin-Related Immune Dysfunction in Health and in Human Immunodeficiency Virus Disease

**DOI:** 10.1155/2008/790309

**Published:** 2008-08-05

**Authors:** Yi Jiang, Pauline E. Jolly, Peter Preko, Jia-Sheng Wang, William O. Ellis, Timothy D. Phillips, Jonathan H. Williams

**Affiliations:** ^1^Department of Epidemiology, School of Public Health, University of Alabama at Birmingham, Birmingham, AL 35226, USA; ^2^St. Markus Hospital and AIDS ALLY, Kumasi, Ghana; ^3^Department of Environmental Toxicology, Texas Tech University, Lubbock, TX 79409, USA; ^4^Department of Biochemistry, Kwame Nkrumah University of Science and Technology, Kumasi, Ghana; ^5^College of Veterinary Medicine, Texas A & M University, College Station, TX 77843, USA; ^6^College of Agricultural and Environmental Sciences, University of Georgia, Griffin, GA 30223, USA

## Abstract

Both aflatoxin and the human immunodeficiency virus (HIV) cause immune suppression and millions of HIV-infected people in developing countries are chronically exposed to aflatoxin in their diets. We investigated the possible interaction of aflatoxin and HIV on immune suppression by comparing immune parameters in 116 HIV positive and 80 aged-matched HIV negative Ghanaians with high (≥0.91 pmol/mg albumin) and low (<0.91 pmol/mg albumin) aflatoxin B1 albumin adduct (AF-ALB) levels. AF-ALB levels and HIV viral load were measured in plasma and the percentages of leukocyte immunophenotypes and cytokine expression were determined using flow cytometry. The cross-sectional comparisons found that (1) among both HIV positive and negative participants, high AF-ALB was associated with lower perforin expression on CD8+ T-cells (*P* = .012); (2) HIV positive participants with high AF-ALB had significantly lower percentages of CD4+ T regulatory cells (Tregs; *P* = .009) and naive CD4+ T cells (*P* = .029) compared to HIV positive participants with low AF-ALB; and (3) HIV positive participants with high AF-ALB had a significantly reduced percentage of B-cells (*P* = .03) compared to those with low AF-ALB. High AF-ALB appeared to accentuate some HIV associated changes in T-cell phenotypes and in B-cells in HIV positive participants.

## 1. INTRODUCTION

Numerous studies conducted in animals and in animal and human cell cultures have shown that aflatoxin exposure can suppress immune function especially cell-mediated immune responses [1, 2]. More specifically, these studies on the immunotoxic effect of aflatoxin have shown that exposure to aflatoxin decreased T or B lymphocyte activity [3, 4], impaired macrophage/neutrophil effector functions [5–8], modified synthesis of inflammatory cytokines [8, 9], suppressed NK cell-mediated cytolysis [10], decreased resistance to infectious diseases [2, 11–15], induced reactivation of chronic infection [16, 17], decreased immunity to vaccination [18, 19], and impaired immune function in developing animals [7, 20].

However, only two studies have been conducted on the immune effects of aflatoxin in humans exposed to low levels of aflatoxin in contaminated foods. One study conducted in Gambian children reported that secretory immunoglobulin A in saliva may be reduced by dietary levels of aflatoxin [21]. We previously reported that the percentages of CD8+ T-cells that expressed perforin, or both perforin and granzyme A were significantly lower in participants with high AFB1-albumin adduct (AF-ALB) levels in plasma compared to those with low AF-ALB [22]. We also found that low levels of CD3+CD69+ and CD19+CD69+ cells were significantly associated with high AF-ALB levels. These alterations in immunological parameters in participants with high AF-ALB levels could result in impairments in cellular immunity in these individuals that could decrease their resistance to infections.

Hendrickse et al. [23] investigated the reasons for the rapid progression of human immunodeficiency virus (HIV) and acquired immune deficiency syndrome (AIDS) in heroin addicts in the Netherlands and Scotland. They found that street heroin was often contaminated with aflatoxin, and that aflatoxin derivatives were commonly found in the body fluids of the addicts. They speculated that the accelerated rate of HIV progression was due to aflatoxin-related immune suppression, but did not undertake studies to examine this. This suggestion of synergy between aflatoxin and HIV progression is also supported by the broad correlation between estimated aflatoxin exposure and the commonly perceived faster rate of HIV progression in Africa than in developed countries in Europe or the United States of America [24, 25]. The HIV pandemic is critical enough for this possibility to be investigated as a matter of urgency.

HIV infection results in impaired immune function that can be measured by changes in immunphenotypically defined lymphocyte subsets and other in vitro functional assays. The altered expression of lymphocyte surface antigens reflects the dynamic interaction between the immune system and HIV. Investigation of the effect of aflatoxin on the immune system in HIV positive individuals is urgently needed since both aflatoxin and HIV are immune suppressive and millions of people who are chronically exposed to aflatoxin in their diet in developing countries are also HIV positive. In this study, we examined the potential immune suppressive interaction of aflatoxin and HIV by measuring a broad array of immune indices in HIV-infected individuals with high and low AF-ALB levels. HIV negative participants were included as a control group.

## 2. MATERIALS AND METHODS

### 2.1. Study design and study participants

This was a cross-sectional analysis of 116 HIV positive individuals and 80 HIV negative control participants. The HIV positive patients and HIV negative controls of comparable age were recruited from the St. Markus Hospital and surrounding communities in Kumasi, Ghana. The protocol for the study was approved by the Institutional Review Board of the University of Alabama at Birmingham (UAB) and the Medical School Ethics Committee of the Kwame Nkrumah University of Science and Technology (KNUST) and participants gave informed consent. The participants were asked to complete a survey on socio-demographic characteristics, and a 20 mL blood sample was collected from each in EDTA vacutainer tubes. Plasma was separated and peripheral blood mononuclear cells (PBMCs) were prepared using ficoll-hypaque density gradients as previously done [26]. PBMCs were stored frozen in liquid nitrogen and shipped to UAB for analysis.

### 2.2. Determination of AF-ALB levels in plasma by radioimmunoassay

AF-ALB levels in plasma of study participants were determined by radioimmunoassay (RIA) as published previously [27]. Briefly, human plasma samples were concentrated by high-speed centrifugal filtration using Microcon-50 microconcentrator with a 50 000 mol. wt. filter cutoff. The concentrated protein was resuspended in 100–150 *μ*L PBS and the amount of human plasma albumin determined in each sample using a bromocresol purple dye binding method. In addition, the amount of total protein was determined by the procedure of Bradford (Pierce Biotechnology Inc., Rockford, IL). Total protein was then digested with Pronase (Calbiochem, La Jolla, Calif, USA; 70 000 proteolytic units/g dry weight was dissolved in PBS at 10 mg/mL) at a ratio of 4 : 1 (Protein:pronase) in a shaking water bath (50 strokes/min) at 37°C for 16–18 hours. Digestion was stopped by cooling on ice. Two volumes ice-cold acetone were added and the sample mixed and allowed to remain at 4°C for 1 hour. The suspension was then centrifuged at 11 000 rpm (9800 g) for 15 minutes. The resulting supernatant containing the bound aflatoxin was decanted and dried in vacuo using a savant speed-vac concentrator. The RIA procedure was used to quantify aflatoxin B1-albumin adducts in duplicate human plasma protein digests each containing 2 mg protein. Nonspecific inhibition in the assay was determined by processing pooled normal human plasma standards obtained from Sigma Aldrich (St. Louis, MO, USA) and the average value of the background was subtracted from the values of test samples in calculating AFB1-albumin adduct levels. The standard curve for the RIA was determined using a nonlinear regression method [28] and values were expressed as the amount of AFB1 per mg albumin [27]. The detection limit of the assay was 0.01 pmol/mg albumin.

### 2.3. Determination of percentages of leukocyte immunophenotypes using flow cytometry

We determined the percentages of leukocyte immunophe-notypes in PBMCs from the study participants. The percentages of T-cells (CD3+), subsets of T-cells (CD4+ and CD8+), B-cells (CD19+), and NK-cells (CD3-CD56+) were measured by flow cytometry. Naive CD4 cells were defined as CD45RA+CD62L+ and memory cells as CD45RO+CD45RA-. The expression of the costimulatory molecule CD28, activation marker HLA-DR, and CD38 on CD4 and CD8 T-cells were also measured. CD8+ T-cell subset classification has been proven useful in monitoring the immune system in several clinical situations [29]. Therefore, we classified CD8+ T-cell subsets into naive (CD8+CD27+CD45RA+), memory (CD8+CD27+CD45RA-), and effector (CD8+CD27-CD45RA+) CD8+ T-cells by flow cytometry. Subtypes of NK-cells, CD3-CD56+CD16-, and CD3-CD56+CD16+ were determined. The percentages of activated (marker CD69) CD3+ T-cells and CD19+ B-cells (CD3+CD69+ and CD19+CD69+), and CD4+CD25+, CD4+CD25+ CD45RO+ T regulatory cell were also measured.

PBMCs were incubated with combinations of fluorescein FITC-, PE-, PerCP-labeled monoclonal antibodies (MAbs) against CD3, CD4, CD8, CD16, CD19, CD25, CD27, CD38, CD45RA, CD45RO, CD56, CD62, CD69, and HLA-DR (BD PharMingen, San Diego, Calif) for 30 minutes at 4°C. Isotype-matched irrelevant FITC-, PE-, and PerCP-labeld MAbs (BD PharMingen, San Diego, Calif) were used as controls in the experiments. After washing the cells three times in PBS, cell fluorescence for each phenotype was analyzed using Becton Dickinson (San Diego, Calif, USA), FACS, and CELLQuest software.

### 2.4. Determination of cytokine expressing CD8+ and CD3-CD56+ cells

CD8+ T-cell cytokine expression (perforin and granzyme A) was measured by intracellular cytokine staining and multiparameter flow cytometry. Also, the cytotoxicity potential of NK-cells was examined by detecting perforin expression in phenotypically defined NK-cells (CD3-CD56+).

For intracellular cytokine staining, PBMCs (1 × 10^6^) were collected in dPBS and washed once with cold dPBS containing 1% BSA. Cells were then resuspended in 100 *μ*L of staining buffer (PBS supplemented with 0.1% sodium azide and 1% FBS pH 7.4) and the phenotypic MAb (CD3, CD8, and CD56) and incubated at 4°C for 30 minutes. After staining, the cells were washed with PBS and resuspended in 1 mL of fix/perm buffer (BD PharMingen, San Diego, Calif). The cells were then fixed for 30 minutes at 4°C, washed, resuspended in 3 mL perm staining buffer and incubated with cytokine antibodies (antiperforin, antigranzyme A) (BD PharMingen, San Diego, Calif) in the presence of 50 uL of permeabilization buffer for 30 minutes at 4°C. The cells were then washed with perm buffer and resuspended in 300 *μ*L of fixative buffer (BD PharMingen) for flow cytometric analysis on a Becton Dicknson FACS using CELLQuest software.

### 2.5. Quantitative viral load assay

HIV RNA was measured using a quantitative reverse transcriptase polymerase chain reaction assay (Amplicor Monitor, Roche Diagnostic System, Brandersburg, NJ, USA). Virus from 0.2 mL of plasma was lysed using the kit lysis buffer and the HIV RNA was precipitated using isopropanol and pelleted by centrifugation. After washing with ethanol, the RNA was resuspended using the kit dilution buffer. Extracted RNA was amplified and detected according to the manufacturer's instructions, and results were reported as HIV RNA copies/mL. All undetectable values (below 400 copies) were assigned a value of 399.

### 2.6. Statistical analysis

Data were entered and analyzed using Windows SPSS version 11.5 (SPSS Inc., Clay, NC, USA). Data are expressed as the means ± SD and the median. For analysis, HIV negative and HIV positive participants were divided into high and low AF-ALB subgroups based on the median AF-ALB level for the group. Groups were compared by intragroup comparisons (high versus low, among either HIV positive or HIV negative participants) and by intergroup comparisons (HIV positive versus HIV negative participants, among either high or low AF-ALB), using Mann-Whitney U-tests. Possible correlates of AF-ALB-associated accelerated HIV disease progression included those indices that were similarly and significantly associated with AF-ALB, as determined by intragroup comparisons among both HIV positive and HIV negative participants, and with HIV infection, as determined by at least 1 intergroup comparison among high and low AF-ALB participants. A probability value of *P* < .05 was considered statistically significant. Correlations using nonparametric methods were also conducted to examine the association between AF-ALB and the immune parameters for the entire group and for HIV positive and HIV negative groups separately.

## 3. RESULTS

### 3.1. Demographic and selected clinical characteristics of participants

Demographic and clinical characteristics for the 116 HIV positive study participants and 80 HIV negative controls are summarized in Table 1. The mean age for the HIV positive group was 38.25 ± 9.44 years and for the HIV negative group was 40.77 ± 17.52 years. Approximately 65% of HIV positive participants were females compared to 44% females in the HIV negative group. AF-ALB levels for the 196 study participants ranged from 0–3.48 pmoL/mg albumin with a mean of 1.01 ± 0.53 and median of 0.91 pmoL/mg albumin. The mean AF-ALB was 1.01 ± 0.61 with median of 0.91 pmoL/mg albumin for the HIV positive group and 1.01 ± 0.41 with median of 0.91 pmoL/mg albumin for the HIV negative control group. Both groups of participants were divided into high AF-ALB (≥0.91)and low AF-ALB (<0.91) subgroups based on the median AF-ALB levels. For HIV positive participants, the mean virus load was higher among the high AF-ALB group compared to the low AF-ALB group (85,049 versus 70,260 copies/mL), but this difference was not statistically significant (*P* = .709). The mean CD4+ T-cell count in the HIV positive participants was 307.46 ± 248.37 cells/*μ*L with a range of 37–1505 cells/*μ*L. The mean CD4+ T-cell count in the HIV negative participants was 1099.04 ± 454.91 cells/*μ*L (range 472–2099 cells/*μ*L). Based on the CDC classification system [30], 17 HIV positive patients were placed in category A, 52 in category B, and 47 in category C.

### 3.2. T, B, and NK cell phenotypes of intergroup and intragroup comparisons

**Figure 1:**
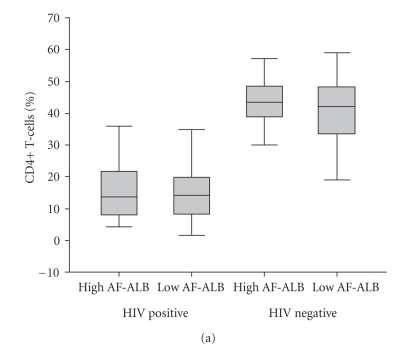

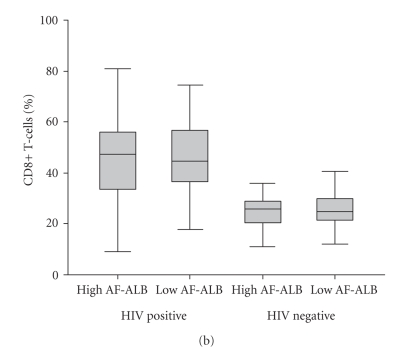

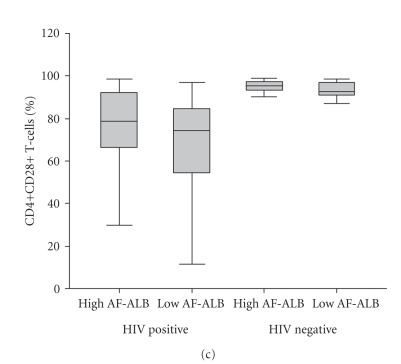

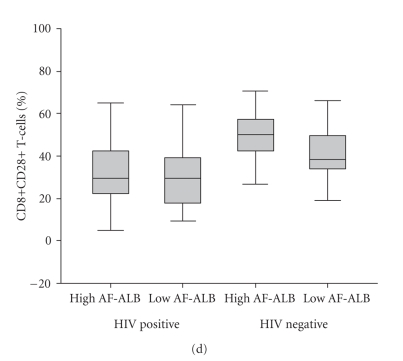

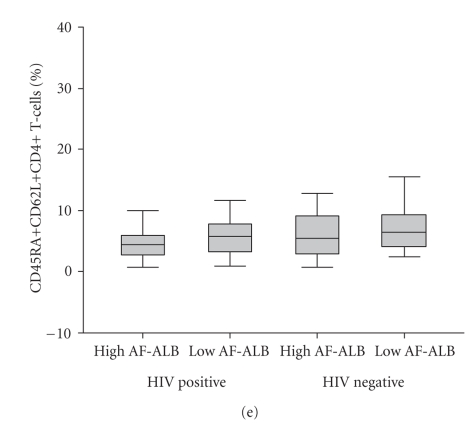

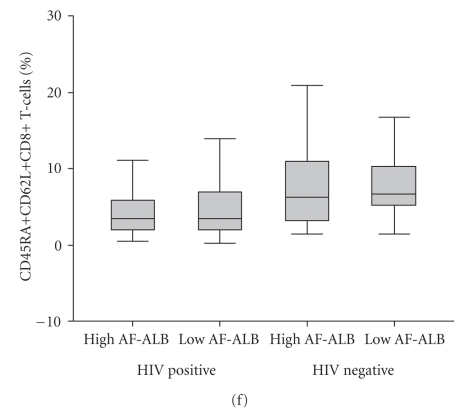

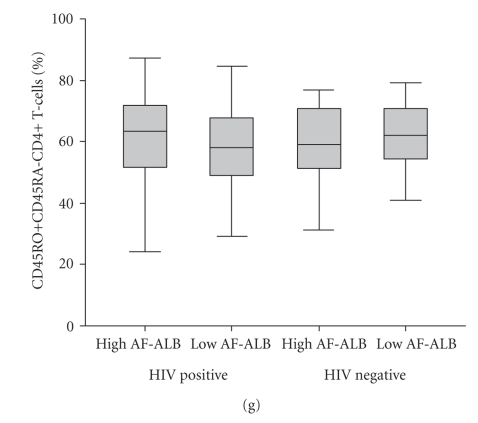

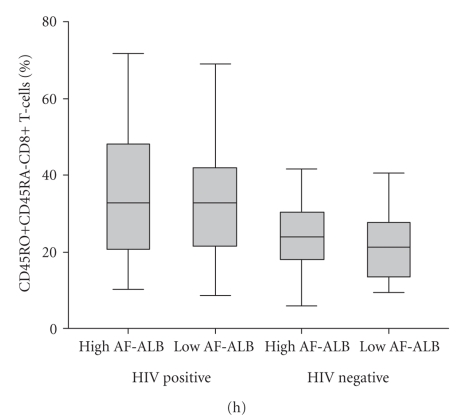

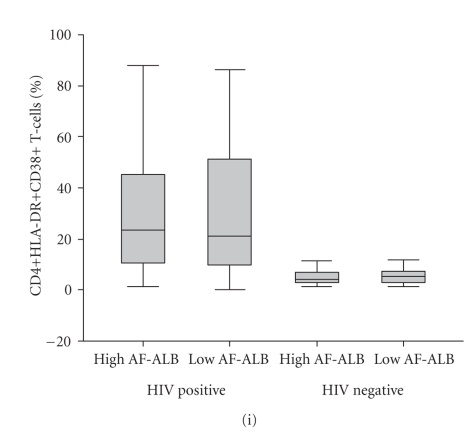

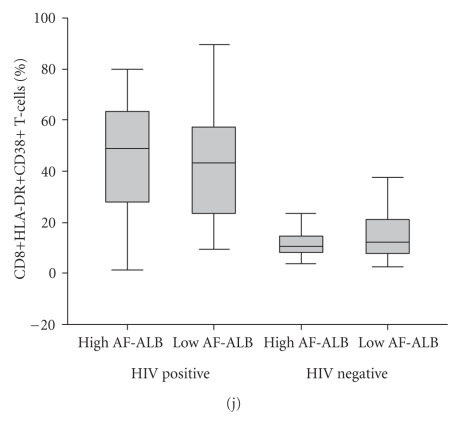

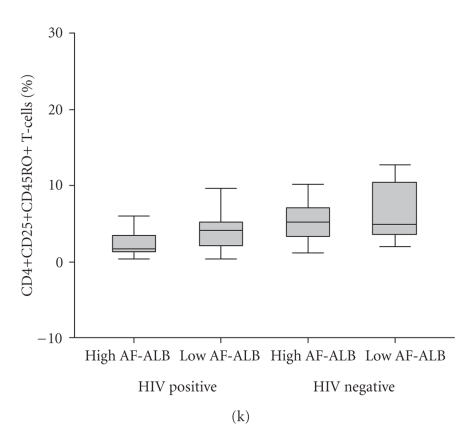

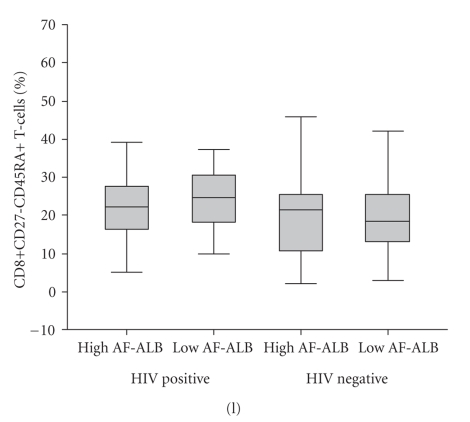

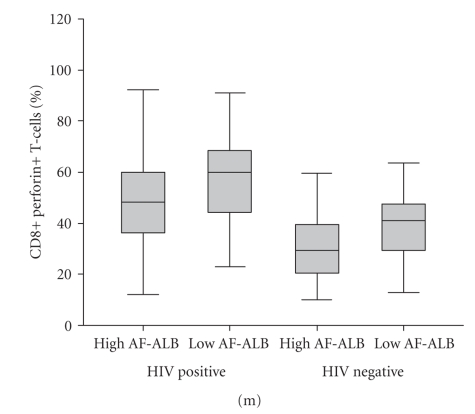

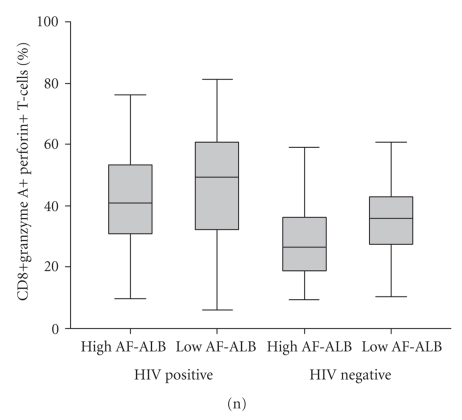

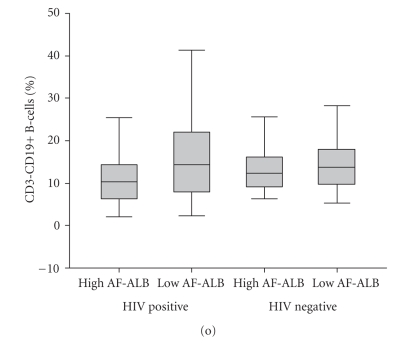

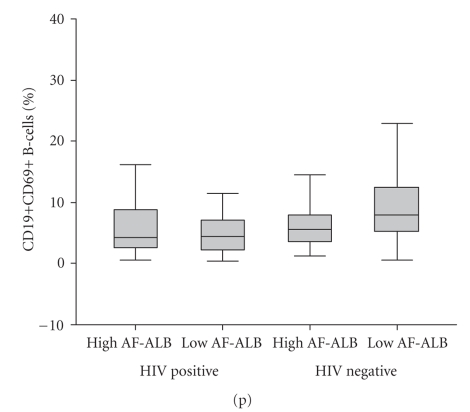

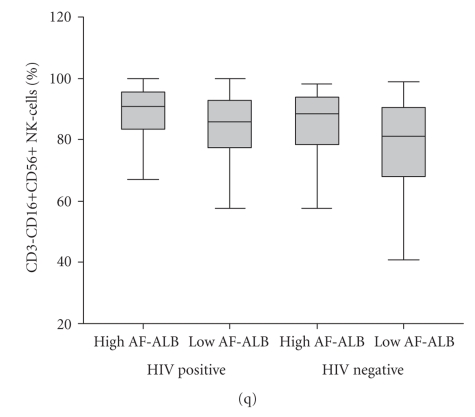

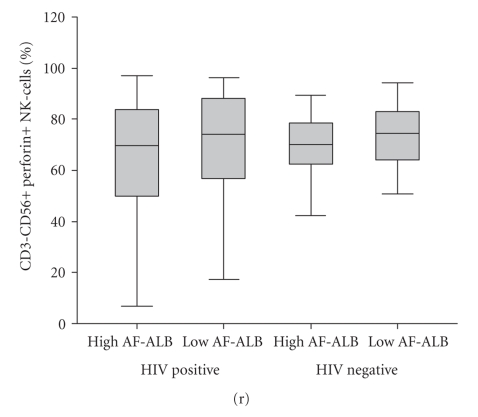
Percentages of T-, B-, and NK-cells in PBMCs in relation to aflatoxin B1 albumin adduct (AF-ALB) levels and HIV infection. The percentages are shown for HIV positive high AF-ALB (*n* = 58) and low AF-ALB (*n* = 58) groups, and HIV negative high AF-ALB (*n* = 40) and low AF-ALB (*n* = 40) groups.

Nominally, significant inter- and intragroup differences are summarized in Figure 1 and Table 2. When intergroup comparisons were made, HIV associated differences were mostly similar among both high AF-ALB and low AF-ALB groups. Relative and absolute CD4+ T-cells were significantly decreased in HIV positive participants compared to HIV negative controls (Figure 1(a)) and the proportions of CD8+ T-cells were higher in HIV positive participants than in the negative controls (*P* = .000, Figure 1(b)). Differences in lymphocyte antigen expression that were evident in the HIV positive group were the CD28+ and HLA-DR+CD38+ percentages within both the CD4+ and CD8+ lymphocyte populations (all *P* ≤ .001, Table 2, Figures 1(c), 1(d), 1(i), and 1(j)). There were fewer CD4+CD25+ and CD4+CD25+CD45RO+ regulatory T-cells in HIV positive participants than HIV negative controls among both high and low AF-ALB groups (Table 2, Figure 1(k). The difference was statistically significant among the high AF-ALB group (*P* = .000) and was not statistically significant among the low AF-ALB group (*P* = .061).

HIV infection was associated with less naive CD8+ (CD45RA+CD62L+CD8+) T-cells (Table 2, Figure 1(f)), and more memory CD8+ (CD45RO+CD45RA-CD8+) T-cells (Table 2, Figure 1(h)) among both high AF-ALB (*P* = .017, *P* = .006) and low AF-ALB groups (*P* = .003, *P* = .002). There were more CD8+CD27-CD45RA+ cells in HIV positive participants than HIV negative controls (Table 2, Figure 1(l)) among the low AF-ALB group (*P* = .038) and less CD8+CD27+CD45RA+ cells among the high AF-ALB group (*P* = .03, Table 2). When CD8+ cells were analyzed for the presence of intracellular perforin and granzyme A without stimulation, we found that the percentages of CD8+ T-cells containing both perforin and granzyme A were statistically significantly higher in HIV positive participants with high AF-ALB (*P* = .000) and low AF-ALB (*P* ≤ .003) compared with HIV negative controls (Table 2, Figures 1(m) and 1(n)).

There was lower CD69 expression on CD19+ B-cells in HIV positive participants among both high and low AF-ALB group (Table 2, Figure 1(p)), but this difference was only statistically significant in the low AF-ALB group (*P* = .000). No significant HIV associated difference in CD3-CD56+ and perforin expressing NK-cells were apparent by intergroup comparison among either the high AF-ALB or low AB-ALB groups. With the exception of CD19+CD69+, CD8+CD27-CD45RA+, and CD8+CD27+CD45RA+ cells, many of the significant T-cell perturbations that were associated with HIV infection in high AF-ALB HIV positive participants were present in their low AF-ALB counterparts.

When intragroup comparisons were made, aflatoxin associated differences depended in part on the HIV serostatus of the participants. Among both the HIV positive and HIV negative participants, higher AF-ALB was associated with lower expression of perforin on CD8+ T-cells (Table 2, Figure 1(m)). Lower perforin and granzyme A expressing CD8+ T-cells also were seen in both groups (Table 2, Figure 1(n)), but the difference was only statistically significant for the HIV negative groups (*P* = .01). Additional aflatoxin associated differences among HIV positive participants included lower percentage of CD4+CD25+CD45RO+ regulatory T-cells (*P* = .009), which was associated with HIV infection in both high and low AF-ALB HIV positive participants (Table 2, Figure 1(k)), and lower percentage of naive CD4+ (CD4+CD45RA+CD62L+) T-cells (Table 2, Figure 1(e)) in the high AF-ALB group.

AF-ALB associated reduction in the B-cells was apparent in HIV positive participants (*P* = .03) but not in HIV negative participants (Table 2, Figure 1(o)). High AF-ALB associated differences among HIV negative controls included less CD69 expression on both CD3+ T-cells (*P* = .024) and CD19+ B-cells (*P* = .027) (Table 2, Figure 1(p)).

No significant aflatoxin-related differences in NK (CD3-CD56+ or CD3-CD56+CD16+) cells and perforin expressing NK (CD3-CD56+Perforin) cells were apparent by intragroup comparisons among either the HIV positive or the HIV negative participants (Table 2, Figures 1(q) and 1(r)).

When we conducted correlation analyses between AF-ALB and the immune parameters among HIV positive participants, we found significant correlations between AF-ALB and perforin-expressing CD8+ T-cells (*r* = −0.170; *P* = .045), T-regulatory cells (*r* = −0.395; *P* = .002), and B-cells (*r* = −0.212; *P* = .012). These findings are similar to our findings above. Also our results were consistent with those presented above for the entire study group and for HIV negative participants.

## 4. DISCUSSION

To identify possible correlates that may underlie the interaction of AF-ALB with HIV disease progression, we sought to identify immune perturbations that are common to both conditions. This study demonstrated, for the first time, associations of aflatoxin with immune parameters in HIV-infected people.

The changes observed in CD3+, CD4+, and CD8+ T-cell phenotypes, CD19+ B-cells and CD3-CD56+ NK-cells in HIV positive compared to HIV negative participants (intergroup comparison) in this study are consistent with previously well-characterized, HIV-associated changes in these cells. HIV-associated immune perturbations were largely similar in participants with high and low AF-ALB levels. HIV infection induced a decrease in CD4+ T- cell numbers and concomitantly activated the immune system. HIV infection was associated with greater expression of HLA-DR/CD38 and lower expression of CD28 on CD4+ and CD8+ T-cells. We found that the surface expression of HLA-DR/CD38 in both CD4+ and CD8+ T-cells was significantly increased in HIV positive participants. The means of CD4+ and CD8+ cells expressing HLA-DR/CD38 progressively increased with advancing clinical disease as determined by CDC stage (data not shown). Also there was a strong negative correlation between both CD4+ T-cell percentage and CD4+ T-cell count with HLA-DR+CD38+ expressing CD4+ T-cells (data is not shown). This activated immune phenotype has been extensively validated in prior studies and demonstrates T-cell activation to be a strong prognostic indicator for progression to AIDS [31–33]. T-cell activation is believed to be the major cause of CD4+ T-cell depletion in HIV infection, through a progression of activation-induced cell death (AICD) [34–37]. The increased immune activation together with increased viral replication causes severe depletion of CD4+ T-cells, eventually leading to the development of AIDS. Therefore, our data suggest that HLA-DR/CD38 could be used as a progression marker in HIV-infected Ghanaians as in HIV-infected North Americans [38].

In the present study, the principal costimulatory molecule CD28 has been uniformly downmodulated in CD4+ and CD8+ T-cells in HIV infected participants. Engagement of the CD28 molecule on CD8+ T lymphocytes in HIV positive individuals during activation has been reported to increase CD8+ T-cell proliferation and differentiation and to prevent apoptosis [39–41]. T-cell receptor stimulation in the absence of CD28 often leads to anergy and to cell death via apoptosis in HIV-infected patients [42]. Many studies have suggested that HIV induces dysfunction of CD4+ and CD8+ cells by CD28 downregulation [43, 44]. The loss of CD28 expression on CD4+ and CD8+ cells in HIV infected participants may be associated with the functional defect of the T-cells and progression to AIDS.

CD8+ T-cells are very important lymphocyte subsets in the immune response against HIV infection. Naive CD8+ T-cells can differentiate into effector-type CD8+ T-cells after they have recognized MHC-matched antigens and then express cytolytic molecules, such as perforin and granzymes which are stored in intracellular granules. Therefore, perforin is expressed in antigen-primed CD8+ T-cells with a cytolytic activity potential that contribute to the inhibition of pathogen spread through immediate lysis of infected cells [45]. In our study, the percentage of naive CD8+ T-cells in HIV infected participants was lower than in HIV negative controls, and the percentage of perforin expressing CD8+ T-cells was significantly increased in the HIV-infected participants than in HIV negative controls. Our observation is consistent with other reports [33, 34]. Overexpression of perforin in HIV-infected participants may be the consequence of CD8+ T-cell hyperactivation and expansion as a part of feedback regulation of anti-HIV cytotoxic T-lymphocyte (CTL) activity [46, 47]. However, among both the HIV infected and HIV negative control groups, those with high AF-ALB showed a lower percentage of perforin expressing CD8+ T-cells compared to those with low AF-ALB levels. This may indicate that CD8+ T-cells synthesizing perforin to enhance the CTL response are impaired in individuals with high AF-ALB [22]. Thus cellular immune function against infectious diseases, such HIV infection will be affected.

T-regulatory cells (Tregs) represent 5–10% of peripheral CD4+ T-cells in healthy individuals and are characterized by constitutive expression of CD25+ and CD45RO+. Tregs (CD4+CD25+CD45RO+) have been implicated in controlling responses to chronic pathogens [48–50] and are known to profoundly inhibit both CD4+ and CD8+ T-cell activation, proliferation, and effector function, although the mechanism of this inhibition remains unclear. Thus Tregs may play a critical role in limiting immunopathology that results from persistent high level immune stimulation from chronic viral infections [51]. We evaluated this population of cells in HIV positive and HIV negative participants for both high and low AF-ALB groups. We found that CD4+CD25+CD45RA+ Tregs showed a tendency to decrease in HIV infected participants with both high and low AF-ALB level compared to HIV negative controls. In addition, we found that the HIV positive group with high AF-ALB had the lowest percentage of Tregs of all the groups suggesting that there is a loss of Tregs in HIV infected participants with high AF-ALB. This loss may facilitate the immune hyperactivation associated with HIV and lead to more severe disease in those with high aflatoxin levels.

The activation molecule CD69 is a costimulatory molecule for lymphocyte proliferation. It is expressed early on the membranes of T- and B-lymphocytes through the stimulated antigen receptor/CD3 complex or cross-linking of surface immunoglobulins, respectively. T-lymphocyte activation via CD69 will progress to proliferation, thus amplifying immune responses [52]. In our present study, the levels of CD69 expression on CD3+ subsets did not differ between the HIV positive and HIV negative control groups. This supports the observation by Krowka et al. and Böhler et al. [53, 54] that unstimulated T-cells from HIV- infected and negative control individuals exhibited similar levels of CD69 expression [53, 54]. The reduced proportions of T-lymphocytes coexpressing CD69 were seen only upon mitogen stimulation in HIV-infected adults [53]. Among HIV negative controls, the significantly decreased percentage of CD69 on CD3+ T-cells was found in the high AF-ALB group compared to the low AF-ALB group as reported before [22]. This could partially contribute to the inability of these cells to mount appropriate immune responses. However, this tendency was not seen when we compared the high AF-ALB group to the low AF-ALB group among HIV positive participants. This finding probably reflects the coexistence of immune activation as shown by increased HLA-DR and CD38 expressing CD4+ and CD8+ T-cells, and immune deficiency as shown by reduced numbers of CD4+ lymphocytes, and impaired T- lymphocyte proliferation in HIV-infected individuals [55]. It has been reported that there is the tendency for CD69 expression on unstimulated peripheral blood T-cells to increase with plasma HIV viral load [56]. Therefore, the variability in plasma HIV viral load level may also contribute to our observation.

In keeping with findings of a previous study [22], we found that high AF-ALB was associated with the reduced percentages of unstimulated CD19+ B-cells coexpressing CD69 in both HIV infected and control groups, but only the difference for the HIV negative groups reached statistical significance. HIV infection was also associated with decreased CD19+CD69+ B-cells in both groups but only the difference for the low AF-ALB group reached statistical significance. No previous data exist concerning the CD69 expressing B-cell in HIV-infected adults. However, there are some reports that have shown that HIV-induced B-cell hyperactivity is associated with reduction of CD21 [57], and CD27 [58, 59] expression in HIV infected patients. Ginaldi et al. [60] reported that B-cells from HIV infected patients show a significantly lower number of HLA-DR molecule per cell compared to normal controls. Our study shows that the ability of B-cell to express CD69 has been impaired in both high AF-ALB exposure and HIV infection. This could suggest that AF-ALB may accentuate the changes in HIV-related immune parameters.

High aflatoxin appeared to accentuate some HIV-associated changes in T-cell phenotypes and B-cells in HIV-infected participants. Because of the cross-sectional design of this study, however, we cannot exclude the possibility that these intragroup differences may simply reflect an imbalance in the severity of HIV disease, with more advanced disease (not evident by our CDC classification) in the high AF-ALB group. However, despite these limitations, we believe that this study has a potentially significant impact on understanding the effects of aflatoxin in HIV disease progression.

A better understanding of the interaction of high aflatoxin with HIV disease might provide insight into key mechanisms that underlie the immunopathogenesis of both processes. Potential immune correlates of this interaction may include reduced Tregs, impaired CD8+ T-cell function, plus impaired B-cell CD69-expressing ability. In HIV-infected patients with higher AF-ALB, loss of Tregs, and persistent immune activation might lead to exhaustion of the naive CD8+ T-cell pool, uniform downmodulated of the principal costimulatory molecule CD28 on CD8+ and CD4+ cells, impaired function of CD8+ T-cells synthesizing perforin (which results in failure of cytolysis by CD8+ T-cells), plus impaired ability of B-cell to express CD69. This will result in increased viral replication and increase in the occurrence of viral escape mutants. Because of viral escape, viral load will increase resulting in further loss of functional HIV specific CD4+ T-cell and a progressive deterioration of the immune system.

Given these observations, high aflatoxin may promote more rapid HIV disease progression in HIV-infected Ghanaians. Our findings should be considered exploratory, given the cross-sectional design of this study and because there may be other variables not considered that may affect immune cell distribution and function. Further clarification of these and other possible immune correlates of the interaction of high aflatoxin with HIV disease progression might be afforded by additional investigation.

## 5. CONCLUSION

High AF-ALB appeared to accentuate some HIV-associated changes in T-cell phenotypes and B-cells in HIV positive participants. These results may indicate that CD8+ T-cells synthesizing perforin to enhance the CTL response are impaired in individuals with high AF- ALB. The loss of Tregs in HIV positive participants with high AF-ALB may facilitate HIV associated immune hyperactivation and lead to more severe disease.

## Figures and Tables

**Table 1:** Demographic and clinical characteristics of HIV positive and HIV negative study participants.

Characteristic	HIV positive		HIV negative	
	High AF-ALB	Low AF-ALB	High AF-ALB	Low AF-ALB
Total number of participants	58	58	40	40

Age (mean ± SD years)	38.28 ± 9.65	38.22 ± 9.30	39.50 ± 18.25	42.08 ± 16.86

Gender number (%)				
Male	24 (41.4)	17 (29.3)	23 (57.5)	22 (55)
Female	34 (58.6)	41 (70.7)	17 (42.5)	18 (45)

Viral load (mean ± SD copies/ml)	85049 ± 230696	70260 ± 193163	0	0
CD4 count (mean ± SD cells/*μ*L)	316 ± 266	298 ± 230	1001 ± 209	1197 ± 614

CDC classification number (%)				
A	10 (17.3)	7 (12.1)		
B	25 (43.1)	27 (46.5)		
C	23 (39.7)	24 (41.4)		

AF-ALB (mean ± SD pmol/mg albumin)	1.44 ± 0.56	0.59 ± 0.24	1.31 ± 0.36	0.70 ± 0.14

Aflatoxin B1 albumin adducts (AF-ALB)—Mean ± SD = 1.01 ± 0.53 pmoL/mg albumin; median = 0.91 pmoL/mg albumin; range = 0–3.48 pmoL/mg albumin. “High AF-ALB” participants were ≥0.91 pmoL/mg albumin; “low AF-ALB” participants were <0.91 pmoL/mg albumin. SD = standard deviation.

**Table 2:** Summary of the immune indices in which significant differences (*P* value) were identified by intra- and intergroup comparisons among high and low aflatoxin B1 albumin adduct (AF-ALB) and HIV positive and HIV negative participants.

Cell subset (percentage)	Intergroup comparison		Intragroup comparison	
	High AF-ALB	Low AF-ALB	HIV positive	HIV negative
	HIV positive versus negative	HIV positive versus negative	High versus Low AF-ALB	High versus Low AF-ALB
Lymphocytes (count)	↓ 0.003	↓ 0.045		
CD4+ cells	↓ 0.000	↓ 0.000		
CD4+ cells (count)	↓ 0.000	↓ 0.000		
CD4+CD28+	↓ 0.000	↓ 0.000		
CD45RA+CD62L+CD4+			↓ 0.029	
CD45RO+CD45RA-CD4+				
CD4+HLA-DR+CD38+	↑ 0.000	↑ 0.000		
CD4+CD25+	↓ 0.001	↓ 0.016		
CD4+CD25+CD45RO+	↓ 0.000	↓ 0.061	↓ 0.009	
CD4+CD25-CD45RO-	↑ 0.000	↑ 0.000		
CD4+CD25-CD45RO+	↑ 0.000	↑ 0.000		
CD8	↑ 0.000	↑ 0.000		
CD8+CD28+	↓ 0.000	↓ 0.000		↑ 0.022
CD45RA+CD62L+CD8+	↓ 0.017	↓ 0.003		
CD45RO+CD45RA-CD8+	↑ 0.006	↑ 0.002		
CD8+HLA-DR+CD38+	↑ 0.000	↑ 0.000		
CD8+CD27-CD45RA+		↑ 0.038		
CD8+CD27+CD45RA+	↓ 0.03			
CD8+CD27-CD45RA-				
CD8+Perforin+	↑ 0.000	↑ 0.000	↓ 0.012	↓ 0.008
CD8+Granzyme A+	↑ 0.000	↑ 0.003		
CD8+Granzyme A+Perforin+	↑ 0.000	↑ 0.002		↓ 0.010
CD3-CD19+			↓ 0.03	
CD19+CD69+		↓ 0.000		↓ 0.027
CD3+				
CD3+CD69+				↓ 0.024
CD3-CD56+				
CD3-CD16+CD56+			↑ 0.052	
CD3+CD56+Perforin				

## References

[1] Bondy GS, Pestka JJ (2000). Immunomodulation by fungal toxins. *Journal of Toxicology and Environmental Health, Part B*.

[2] Pier AC, Richard JL, Thurston JR (1986). Immunomodulation in aflotoxicosis. *Diagnosis of Mycotoxicosis*.

[3] Reddy RV, Taylor MJ, Sharma RP (1987). Studies of immune function of CD-1 mice exposed to aflatoxin B_1_. *Toxicology*.

[4] Richard J, Thurston JR, Pier AC, Rosenberg P (1978). Effects of mycotoxins on immunity. *Toxins: Animal, Plant and Microbial*.

[5] Neldon-Ortiz DL, Qureshi MA (1992). Effects of AFB_1_ embryonic exposure on chicken mononuclear phagocytic cell functions. *Developmental & Comparative Immunology*.

[6] Cusumano V, Rossano F, Merendino RA (1996). Immunobiological activities of mould products: functional impairment of 
human monocytes exposed to aflatoxin B_1_. *Research in Microbiology*.

[7] Silvotti L, Petterino C, Bonomi A, Cabassi E (1997). Immunotoxicological effects on piglets of feeding sows diets containing aflatoxins. *The Veterinary Record*.

[8] Moon E-Y, Rhee D-K, Pyo S (1999). In vitro suppressive effect of aflatoxin B_1_ on murine peritoneal macrophage functions. *Toxicology*.

[9] Jakab GJ, Hmieleski RR, Zarba A, Hemenway DR, Groopman JD (1994). Respiratory aflatoxicosis: suppression of pulmonary and systemic host defenses in rats and mice. *Toxicology and Applied Pharmacology*.

[10] Reddy RV, Sharma RP (1989). Effects of aflatoxin B_1_ on murine lymphocytic functions. *Toxicology*.

[11] Hamilton PB, Harris JR (1971). Interaction of aflatoxicosis with *Candida albicans* infections and other stresses in chickens. *Poultry Science*.

[12] Edds GT, Nair KPC, Simpson CF (1973). Effect of aflatoxin B_1_ on resistance in poultry against cecal coccidiosis and Marek's disease. *American Journal of Veterinary Research*.

[13] Wyatt RD, Ruff MD, Page RK (1975). Interaction of aflatoxin with *Eimeria tenella* infection and monensin in young broiler 
chickens. *Avian Diseases*.

[14] Cysewski SJ, Wood RL, Pier AC, Baetz AL (1978). Effects of aflatoxin on the development of acquired immunity to swine erysipelas. *American Journal of Veterinary Research*.

[15] Joens LA, Pier AC, Cutlip RC (1981). Effects of aflatoxin consumption on the clinical course of swine dysentery. *American Journal of Veterinary Research*.

[16] Venturini MC, Quiroga MA, Risso MA (1996). Mycotoxin T-2 and aflatoxin B_1_ as immunosuppressants in mice chronically 
infected with *Toxoplasma gondii*. *Journal of Comparative Pathology*.

[17] Kubena LF, Bailey RH, Byrd JA (2001). Cecal volatile fatty acids and broiler chick susceptibility to *Salmonella typhimurium* 
colonization as affected by aflatoxins and T-2 toxin. *Poultry Science*.

[18] Gabal MA, Azzam AH (1998). Interaction of aflatoxin in the feed and immunization against selected infectious diseases in poultry. II. Effect on one-day-old layer chicks simultaneously vaccinated against Newcastle disease, infectious bronchitis and infectious 
bursal disease. *Avian Pathology*.

[19] Gabal MA, Dimitri RA (1998). Humoral immunosuppressant activity of aflatoxin ingestion in rabbits measured by response to 
*Mycobacterium bovis* 
antigens using enzyme-linked immunosorbent assay and serum protein electrophoresis. *Mycoses*.

[20] Pier AC, McLoughlin ME, Richard JL, Baetz AL, Dahlgren RR, Lacey J (1984). *In utero* transfer of aflatoxin and selected effects on neonatal pigs. *Trichothecenes and Other Mycotoxins*.

[21] Turner PC, Moore SE, Hall AJ, Prentice AM, Wild CP (2003). Modification of immune function through exposure to dietary aflatoxin in Gambian children. *Environmental Health Perspectives*.

[22] Jiang Y, Jolly PE, Ellis WO, Wang J-S, Phillips TD, Williams JH (2005). Aflatoxin B1 albumin adduct levels and cellular immune status in Ghanaians. *International Immunology*.

[23] Hendrickse RG, Maxwell SM, Young R (1989). Aflatoxins and heroin. *British Medical Journal*.

[24] Jaffar S, Grant AD, Whitworth J, Smith PG, Whittle H (2004). The natural history of HIV-1 and HIV-2 infections in adults in Africa: a literature review. *Bulletin of the World Health Organization*.

[25] Morgan D, Whitworth JAG (2001). The natural history of HIV-1 infection in Africa. *Nature Medicine*.

[26] Jolly PE (1997). Replicative characteristics of primary isolates of the human immunodeficiency virus type 1 in peripheral blood mononuclear cells, primary macrophages and CD4^+^ transformed T-cell lines. *Cellular and Molecular Biology*.

[27] Wang J-S, Qian G-S, Zarba A (1996). Temporal patterns of aflatoxin-albumin 
adducts in hepatitis B surface antigen-positive and antigen-negative residents of Daxin, Qidong County, 
People's Republic of China. *Cancer Epidemiology, Biomarkers & Prevention*.

[28] Gange SJ, Muñoz A, Wang J-S, Groopman JD (1996). Variability of molecular biomarker measurements from nonlinear calibration curves. *Cancer Epidemiology, Biomarkers & Prevention*.

[29] Hamann D, Baars PA, Rep MHG (1997). Phenotypic and functional separation of memory and effector human CD8^+^ T cells. *Journal of Experimental Medicine*.

[30] CDC 1993 revised classification system for HIV infection and expanded surveillance case definition for AIDS among adolescents and adults.

[31] Giorgi JV, Lyles RH, Matud JL (2002). Predictive value of immunologic and virologic markers after long or short duration of HIV-1 infection. *Journal of Acquired Immune Deficiency Syndromes*.

[32] Hazenberg MD, Otto SA, van Benthem BHB (2003). Persistent immune activation in HIV-1 infection is associated with progression to AIDS. *AIDS*.

[33] Giorgi JV, Hultin LE, McKeating JA (1999). Shorter survival in advanced human immunodeficiency virus type 1 infection is more closely associated with T lymphocyte activation than with plasma virus burden or virus chemokine coreceptor usage. *Journal of Infectious Diseases*.

[34] Sousa AE, Carneiro J, Meier-Schellersheim M, Grossman Z, Victorino RMM (2002). CD4 T cell depletion is linked directly to immune activation in the pathogenesis of 
HIV-1 and HIV-2 but only indirectly to the viral load. *The Journal of Immunology*.

[35] Hazenberg MD, Hamann D, Schuitemaker H, Miedema F (2000). T cell depletion in HIV-1 infection: how CD4^+^ T cells go out of stock. *Nature Immunology*.

[36] Grossman Z, Meier-Schellersheim M, Sousa AE, Victorino RMM, Paul WE (2002). CD4^+^ T-cell depletion in HIV infection: are we closer to understanding the cause?. *Nature Medicine*.

[37] Anderson RW, Ascher MS, Sheppard HW (1998). Direct HIV cytopathicity cannot account for CD4 decline in AIDS in the presence of homeostasis: a worst-case dynamic analysis. *Journal of Acquired Immune Deficiency Syndromes & Human Retrovirology*.

[38] Giorgi JV, Liu Z, Hultin LE, Cumberland WG, Hennessey K, Detels R (1993). Elevated levels of CD38^+^CD8^+^ T cells in HIV infection add to the prognostic value of low CD4^+^ T cell levels: results of 6 years of follow-up. The Los Angeles Center, Multicenter AIDS Cohort Study. *Journal of Acquired Immune Deficiency Syndromes*.

[39] Brinchmann JE, Dobloug JH, Heger BH, Haaheim LL, Sannes M, Egeland T (1994). Expression of costimulatory molecule CD28 on T cells in human immunodeficiency virus type 1 infection: functional and clinical correlations. *The Journal of Infectious Diseases*.

[40] June CH, Bluestone JA, Nadler LM, Thompson CB (1994). The B7 and CD28 receptor families. *Immunology Today*.

[41] Lewis DE, Ng Tang DS, Adu-Oppong A, Schober W, Rodgers JR (1994). Anergy and apoptosis in CD8^+^ T cells from HIV-infected persons. *The Journal of Immunology*.

[42] Borthwick NJ, Bofill M, Gombert WM (1994). Lymphocyte activation in HIV-1 infection. II. Functional defects of CD28- T cells. *AIDS*.

[43] Choremi-Papadopoulou H, Viglis V, Gargalianos P, Kordossis T, Iniotaki-Theodoraki A, Kosmidis J (1994). Downregulation of CD28 surface antigen on CD4^+^ and CD8^+^ T lymphocytes during HIV-1 infection. *Journal of Acquired Immune Deficiency Syndromes*.

[44] Saukkonen JJ, Kornfeld H, Berman JS (1993). Expansion of a CD8^+^ CD28-cell population in the blood and lung of HIV-positive patients. *Journal of Acquired Immune Deficiency Syndromes*.

[45] Liu C-C, Walsh CM, Young JD-E (1995). Perforin: structure and function. *Immunology Today*.

[46] Portales P, Reynes J, Rouzier-Panis R, Baillat V, Clot J, Corbeau P (2003). Perforin expression in T cells and virological response to PEG-interferon alpha2b in HIV-1 infection. *AIDS*.

[47] Qi W, Yongjun J, Yanan W (2006). Differential expression of perforin in cytotoxic lymphocyte in HIV/AIDS patients of China. *Journal of Clinical Immunology*.

[48] Belkaid Y, Piccirillo CA, Mendez S, Shevach EM, Sacks DL (2002). CD4^+^CD25^+^ regulatory T cells control *Leishmania major* persistence and immunity. *Nature*.

[49] Suvas S, Kumaraguru U, Pack CD, Lee S, Rouse BT (2003). CD4^+^CD25^+^ T cells regulate virus-specific primary and memory CD8^+^ T cell responses. *The Journal of Experimental Medicine*.

[50] Mittrücker H-W, Kaufmann SHE (2004). Mini-review: regulatory T cells and infection: suppression revisited. *European Journal of Immunology*.

[51] Sakaguchi S (2003). Control of immune responses by naturally arising CD4^+^ regulatory T cells that express toll-like receptors. *The Journal of Experimental Medicine*.

[52] Rutella S, Rumi C, Lucia MB (1999). Induction of CD69 antigen on normal CD4^+^ and CD8^+^ lymphocyte subsets and its relationship with the phenotype of responding T-cells. *Cytometry Part B: Clinical Cytometry*.

[53] Krowka JF, Cuevas B, Maron DC, Steimer KS, Ascher MS, Sheppard HW (1996). Expression of CD69 after in vitro stimulation: a rapid method for quantitating impaired lymphocyte responses in HIV-infected individuals. *Journal of Acquired Immune Deficiency Syndromes & Human Retrovirology*.

[54] Böhler T, Walcher J, Hölzl-Wenig G (1999). Expression of CD69 on T-cells from HIV-1-infected children and adolescents increases with increasing 
viral load. *European Journal of Pediatrics*.

[55] De Martino M, Rossi ME, Azzari C (1999). Viral load and CD69 molecule expression on freshly isolated and cultured mitogen-stimulated lymphocytes of children with perinatal HIV-1 infection. *Clinical & Experimental Immunology*.

[56] Fahey JL, Taylor JMG, Manna B (1998). Prognostic significance of plasma markers of immune activation, HIV viral load and CD4 
T-cell measurements. *AIDS*.

[57] Moir S, Malaspina A, Ogwaro KM (2001). HIV-1 induces phenotypic and functional perturbations of B cells in chronically infected individuals. *Proceedings of the National Academy of Sciences of the United States of America*.

[58] Widney D, Gundapp G, Said JW (1999). Aberrant expression of CD27 and soluble CD27 (sCD27) in HIV infection and in AIDS-associated 
lymphoma. *Clinical Immunology*.

[59] De Milito A, Morch C, Sonnerborg A, Chiodi F (2001). Loss of memory (CD27) B lymphocytes in HIV-1 infection. *AIDS*.

[60] Ginaldi L, De Martinis M, D'Ostilio A, Marini L, Quaglino D (1998). Changes in antigen expression on B lymphocytes during HIV infection. *Pathobiology*.

